# Growth Hormone Mediates Pubertal Skeletal Development Independent of Hepatic IGF-1 Production

**DOI:** 10.1002/jbmr.265

**Published:** 2010-10-06

**Authors:** Hayden-William Courtland, Hui Sun, Mordechay Beth-On, Yingjie Wu, Sebastien Elis, Clifford J Rosen, Shoshana Yakar

**Affiliations:** 1 Division of Endocrinology, Diabetes and Bone Diseases, Mount Sinai School of MedicineNew York, NY, USA; 2 Maine Medical Center Research InstituteScarborough, ME, USA

**Keywords:** IGF-1, BONE, LID MICE, MICRO–COMPUTED TOMOGRAPHY, ENDOCRINE, MECHANICAL PROPERTIES

## Abstract

Deficiencies in either growth hormone (GH) or insulin-like growth factor 1 (IGF-1) are associated with reductions in bone size during growth in humans and animal models. Liver-specific IGF-1-deficient (LID) mice, which have 75% reductions in serum IGF-1, were created previously to separate the effects of endocrine (serum) IGF-1 from autocrine/paracrine IGF-1. However, LID mice also have two- to threefold increases in GH, and this may contribute to the observed pubertal skeletal phenotype. To clarify the role of GH in skeletal development under conditions of significantly reduced serum IGF-1 levels (but normal tissue IGF-1 levels), we studied the skeletal response of male LID and control mice to GH inhibition by pegvisomant from 4 to 8 weeks of age. Treatment of LID mice with pegvisomant resulted in significant reductions in body weight, femur length (Le), and femur total area (Tt.Ar), as well as further reductions in serum IGF-1 levels by 8 weeks of age, compared with the mean values of vehicle-treated LID mice. Reductions in both Tt.Ar and Le were proportional after treatment with pegvisomant. On the other hand, the relative amount of cortical tissue formed (RCA) in LID mice treated with pegvisomant was significantly less than that in both vehicle-treated LID and control mice, indicating that antagonizing GH action, either directly (through GH receptor signaling inhibition) or indirectly (through further reductions in serum/tissue IGF-1 levels), results in disproportionate reductions in the amount of cortical bone formed. This resulted in bones with significantly reduced mechanical properties (femoral whole-bone stiffness and work to failure were markedly decreased), suggesting that compensatory increases of GH in states of IGF-1 deficiency (LID mice) act to protect against a severe inhibition of bone modeling during growth, which otherwise would result in bones that are too weak for normal and/or extreme loading conditions. © 2011 American Society for Bone and Mineral Research.

## Introduction

Hormonal action through the growth hormone/insulin-like growth factor 1 (GH/IGF-1) axis is an important regulator of bone formation during growth. Secretion of GH stimulates IGF-1 production in the liver and regulates serum as well as tissue IGF-1 levels. Animal models have shown that gene ablation of IGF-1 results in reduced bone size and decreased bone mineral density (BMD).(1–3) In order to separate the effects of circulating (endocrine) IGF-1 from tissue (autocrine/paracrine) IGF-1, we previously created a liver-specific IGF-1 deficient (LID) mouse model that has 75% reductions in serum IGF-1 levels. Skeletal analysis of male LID mice throughout development revealed impairment of transverse bone growth; beginning as early as 8 weeks and lasting into adulthood, bones were more slender with significant reductions in the amount of cortical bone.(4) However, reductions in serum IGF-1 levels in LID mice were accompanied by two- to threefold elevations in serum GH levels. Thus the possibility exists that GH may regulate skeletal development independent of hepatic IGF-1 (serum) via its binding to the growth hormone receptor (GHR) in bone.

Numerous studies exist demonstrating the importance of GH in mediating skeletal development. For example, growth hormone receptor knockout (GHRKO) mice have decreased femoral length, as well as decreased BMD, resulting from reduced cortical bone area.(5) Conversely, in human acromegaly, excessive GH secretion has been linked to increased BMD(6) and is associated with a decrease risk of fracture.(7) The proper regulation of GH action has implications not only for growth but also for aging and fracture risk. Specifically, GH-deficient patients were found to have up to threefold increases in fracture frequency compared with controls.(8,9) In addition, there is evidence to support the idea that the age-specific onset of GH deficiency may influence fracture risk.(10) Although this literature demonstrates the importance of GH in skeletal maintenance, GH stimulates IGF-1 production, and the aforementioned studies did not clarify what portion of GH's effect is exerted through secondary increases in serum IGF-1 levels and what effect, if any, is exerted directly through GH action via the GHR.

To elucidate the contribution of compensatory increases in GH on bone development in the presence of reduced serum IGF-1 levels, we inhibited GH action in male LID mice by using the growth hormone antagonist pegvisomant during pubertal growth. Pegvisomant, by virtue of its G120K mutation, prevents dimerization of the GHR and therefore all downstream signaling.(11,12) Pegvisomant has been well established as a clinical approach to normalize serum IGF-1 levels in patients with acromegaly,(13) and it is also known to normalize markers of bone formation and resorption.(14,15) By administering pegvisomant to LID mice, which have significantly reduced serum IGF-1 levels, the contribution of GH to skeletal development can be assessed independent of changes in hepatic IGF-1 production.

## Methods

### Animals

Male liver-specific IGF-1-deficient (LID) and control (*Igf1*^floxed^) mice (FVB/N background) were housed two to five per cage, subjected to10-hour light/dark cycles, and given water and food *ad libitum*. Mice were treated with either vehicle (saline) or a growth hormone antagonist pegvisomant (Somavert, Pfizer, Inc., New York, NY, USA) three times a week for 4 weeks beginning at 4 weeks of age. Previously reported doses for pegvisomant treatment have varied between approximately 0.5 mg/mouse per day(16) and approximately 5 mg/mouse per day.(17) For chronic treatment with pegvisomant, we chose a dose of 2 mg/mouse per day. Verification of this dose's ability to inhibit GH action in the liver was confirmed by a reduction in serum IGF-1 levels in control mice, as well as reductions in *Igf1* and the *acid-labile subunit* (*Als*) gene expression in liver 8 hours following pegvisomant injection. After 4 weeks of treatment (8 weeks old), mice were euthanized for phenotypic analysis. The pubertal period (4 to 8 weeks) was chosen for this investigation because during this time serum GH levels peak and rapid changes in femoral size and shape occur for both LID and control mice.(4) All animals were labeled with calcein (10 mg/kg) 7 and 2 days before being euthanized. All procedures involving mice were reviewed and approved by the Institutional Animal Care and Use Committee of the Mount Sinai School of Medicine.

### Serum parameters and gene expression

Serum IGF-1 levels were measured using a commercial radioimmunoassay as described previously.(18–20) Serum osteocalcin was measured using a commercial radioimmunoassay (American Laboratory Products Company, Inc., Salem, NH, USA). For *Igf1* and *Als* gene expression, total RNA was extracted from liver samples of control mice treated with vehicle or pegvisomant using TRIzol in accordance with the manufacturer's instructions (Invitrogen Corp., Carlsbad, CA, USA). After verification of RNA integrity using a Bioanalyzer (2100 Bioanalyzer-Bio Sizing, Version A.02.12 SI292, Agilent Technologies, Santa Clara, CA, USA), 1 µg of RNA was reverse transcribed to cDNA using oligo(dT) primers with an RT-PCR kit (Invitrogen Corp.). Real-time PCR analysis was performed using the same primers.

### Body composition

Body composition (fat and lean mass) was assessed in live (nonanesthetized) animals using MRI (EchoMRI 3-in-1, Echo Medical Systems, LLC, Houston, TX, USA). This technique allowed serial measurements with high levels of precision. The measurement of each mouse lasted 90 seconds, and the precision of the measurements was between 0.1 and 0.3 SDs.

### Bone morphology and tissue mineral density

Femoral morphology of 8-week-old animals was assessed at an 8.7-µm voxel resolution using an eXplore Locus SP Pre-Clinical Specimen Micro–Computed Tomography System (GE Healthcare, London, Ontario, Canada), as described previously.(21) Reconstructed images of mid-diaphyseal cortical bone (2-mm region distal to the third trochanter) and distal metaphyseal bone (2-mm region proximal to the growth plate) were individually thresholded using a standard thresholding algorithm that segmented bone from nonbone voxels. Reconstructed whole-bone images also were obtained for measurement of total femoral length (Le). Cortical parameters measured included total area (Tt.Ar), cortical area (Ct.Ar), marrow area (Ma.Ar), cortical thickness (Ct.Th), and polar moment of inertia (*J*_*o*_). In addition, we measured robustness, which was defined as Tt.Ar/Le and indicates the extent of transverse bone size relative to longitudinal bone size; smaller Tt.Ar/Le values indicate more slender bones. We also measured relative amount of cortical tissue formed (RCA), defined as Ct.Ar/Tt.Ar (a measure of how much bone tissue exists in the total area of bone). The measured trabecular parameters included bone volume fraction (BV/TV), trabecular thickness (Tb.Th), trabecular number (Tb.N), and trabecular spacing (Tb.Sp). Finally, for both cortical and trabecular bone, tissue mineral density (TMD), which represents the average mineral value of bone voxels expressed in hydroxyapatite (HA) density equivalents, was calculated by converting bone voxel grayscale values to mineral values (mg/cc of HA) through the use of a calibration phantom containing air, water, and hydroxyapatite (SB3, Gamex RMI, Middleton, WI, USA).

### Cortical bone histomorphometry

For dynamic histomorphometric measurements, right femurs of 8-week-old animals were cleaned and embedded in polymethyl methacrylate (PMMA) as described previously.(22) Embedded samples were sectioned (200 µm thickness) at the mid-diaphysis, immediately distal to the third trochanter, using a low-speed diamond-coated wafering saw (Buehler, Lake Bluff, IL, USA). Sections were adhered to glass slides using Eukitt's mounting medium, and images of each section (5 × magnification) were obtained using a digital camera (Sony Exwave HAD, 3CCD Camera, Sony, New York, NY, USA) attached to a fluorescence microscope (Zeiss Axioplan2, Zeiss, Thornwood, NY, USA) with a pixel resolution of 2.1 µm. Bone formation was quantified separately for the endosteal and periosteal surfaces. Labeled surface (L.Pm/B.Pm, %), mineral apposition rate (MAR, µm/day), and bone-formation rate (BFR/B.Pm, µm/day × 100) were measured using OsteoMeasure software (Osteometrics, Atlanta, GA, USA), as defined previously.(23)

### Mechanical testing

After imaging by micro–computed tomography (µCT), left femurs from 8-week-old male mice were loaded to failure in four-point bending at 0.05 mm/s using a servohydraulic materials testing system (Instron Corp, Canton, MA, USA) following previously established protocols.(24) Load and midspan deflection were acquired directly by an A/D system at a sampling frequency of 25 Hz. All mechanical testing was performed at room temperature, and femur samples were kept moist with a calcium-supplemented phosphate-buffered saline solution. Load-deflection curves were analyzed for stiffness (the slope of the initial portion of the curve), maximum load (the load at breaking), postyield deflection (PYD), and work to failure (work). PYD, a measure of bone ductility, was defined as the deflection at failure minus the deflection at yield. Yield was defined as a 10% reduction of secant stiffness (load range normalized for deflection range) relative to the initial (tangent) stiffness. Work was defined as the area under the load-deflection curve.

### Statistical analysis

Means and SDs for each trait were determined separately for the treatment groups of each strain. Significant differences in mean trait values of vehicle- and pegvisomant-treated animals of each strain were identified using ANOVA and Bonferroni post-hoc tests (*p* < .05) (Statistica 6.0, Statsoft, Inc., Tulsa, OK, USA). In the case of serum IGF-1 levels, the data were log-transformed before performing ANOVA to account for differences in sample variance.

## Results

### Pharmacologic inhibition of GH action reduces body size and alters body composition

**Fig. 1:**
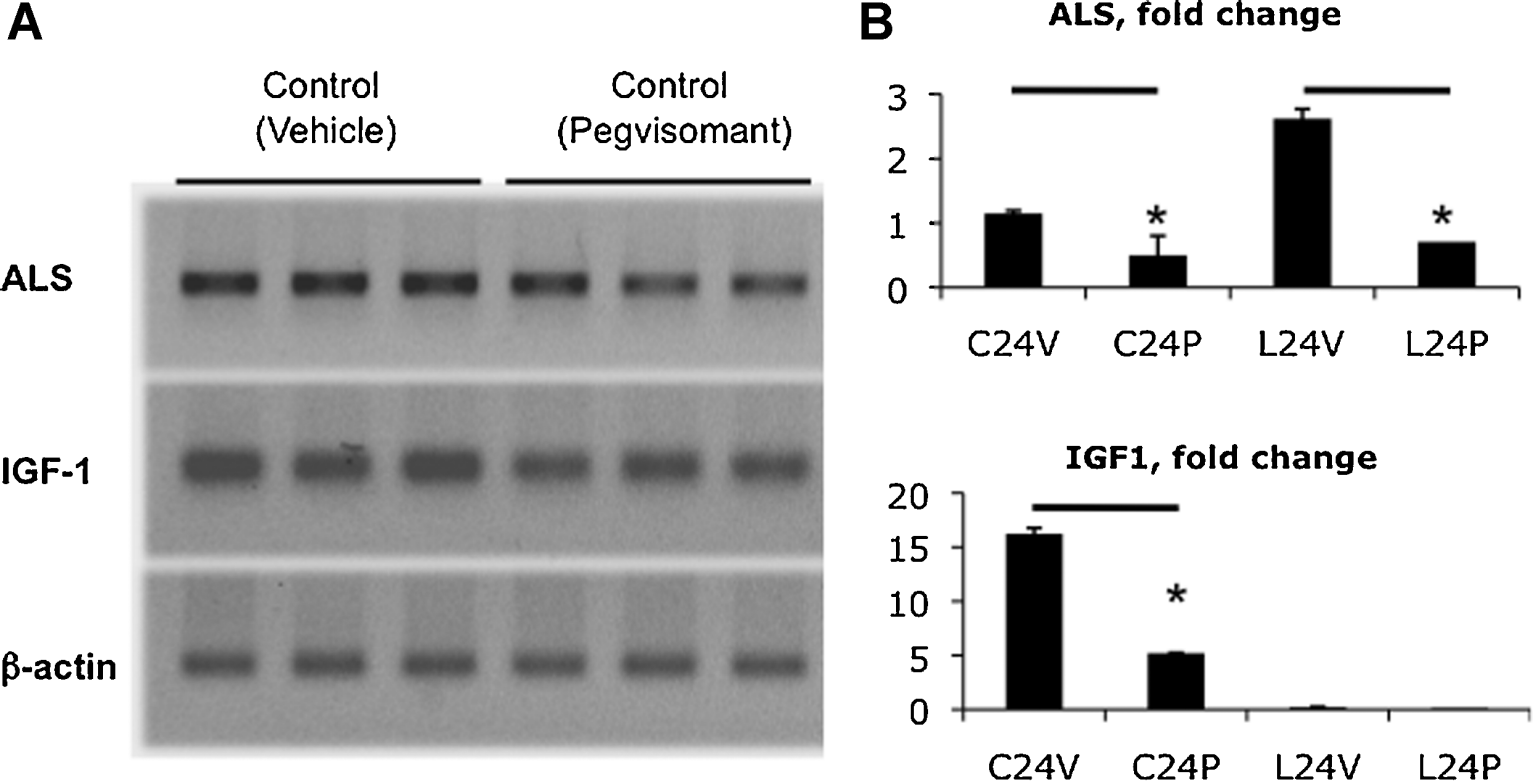
Gene expression studies were performed on mRNA extracted from livers of control and LID mice treated with vehicle or pegvisomant. (*A*) RT-PCR of *Als* and *Igf1* in control livers 8 hours following pegvisomant injection indicated reductions when compared with vehicle-treated controls (β*-actin* served as a loading control). (*B*) Real-time PCR analysis of *Als* and *Igf1* 24 hours following pegvisomant injection showed significant reductions in control mice. *Igf1* gene expression was undetectable in LID mice, whereas *Als* gene expression was inhibited following pegvisomant injection.

**Fig. 2:**
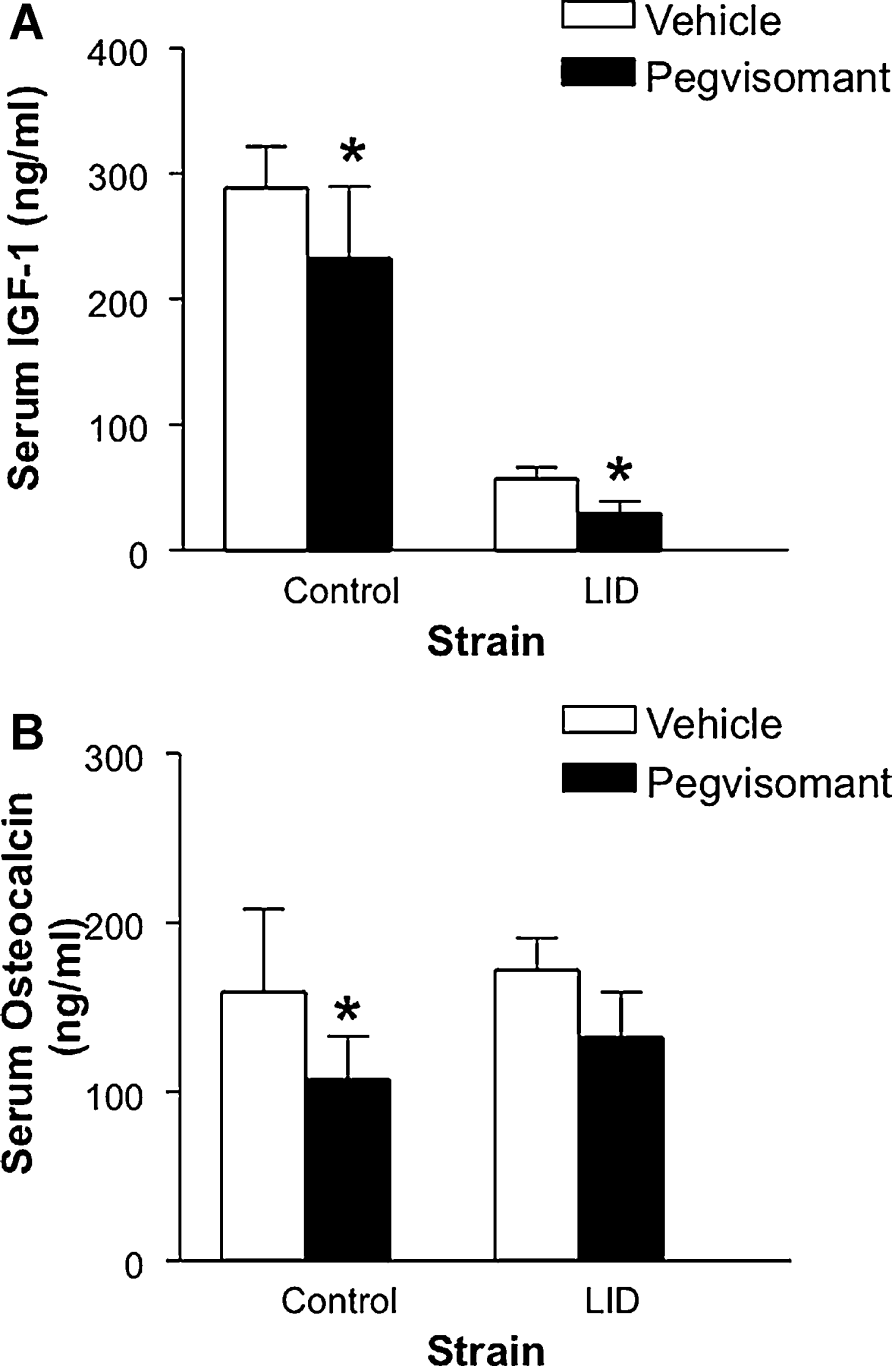
(*A*) Mean serum IGF-1 levels (± SD) for 8-week-old male control and LID mice treated with vehicle (*n* = 15) or pegvisomant (*n* = 11). (*B*) Mean serum osteocalcin levels (± SD) for 8-week-old male control mice treated with vehicle (*n* = 10) or pegvisomant (*n* = 9) and LID mice treated with vehicle (*n* = 9) or pegvisomant (*n* = 10). ^*^Significant difference between vehicle and pegvisomant treatment, ANOVA, *p* < .05.

For this investigation, the pubertal period(4 to 8 weeks) was chosen because during this time, serum GH levels peak and rapid changes in femoral size and shape occur for both LID and control mice.(4) To confirm that our selected dose of pegvisomant successfully blocked GH action, we measured *Als* (a surrogate marker for GH action) and *Igf1* mRNA levels in control mice treated with vehicle or pegvisomant. Eight hours after injection (Fig. 1*A*) and 24 hours after injection (Fig. 1*B*), control mice treated with pegvisomant had significant reductions in *Igf1* and *Als* mRNA levels. LID mice, whether treated with vehicle or treated with pegvisomant, showed undetectable levels of liver *Igf1* gene expression (as expected owing to gene recombination). In LID mice treated with vehicle, in which GH levels are elevated, we could detect elevations in *Als* gene expression. When treated with pegvisomant, *Als* gene expression in LID mice was reduced significantly (Fig. 1*B*). These data are in accordance with previously reported reductions in *Igf1* mRNA levels after acute treatment of mice with pegvisomant.(17) As further confirmation of pharmacologic inhibition, we found that treatment of control mice with pegvisomant resulted in significant reductions in serum IGF-1 levels by 8 weeks of age. Interestingly, in LID mice, where the liver *Igf1* gene is ablated, serum IGF-1 levels after pegvisomant treatment (29 ± 10 ng/mL) also were found to be statistically different from vehicle-treated mice (57 ± 9 ng/mL; Fig. 2*A*), suggesting that GH-dependent IGF-1 secretion from other tissues (muscle or fat) also was inhibited by pegvisomant.

**Fig. 3:**
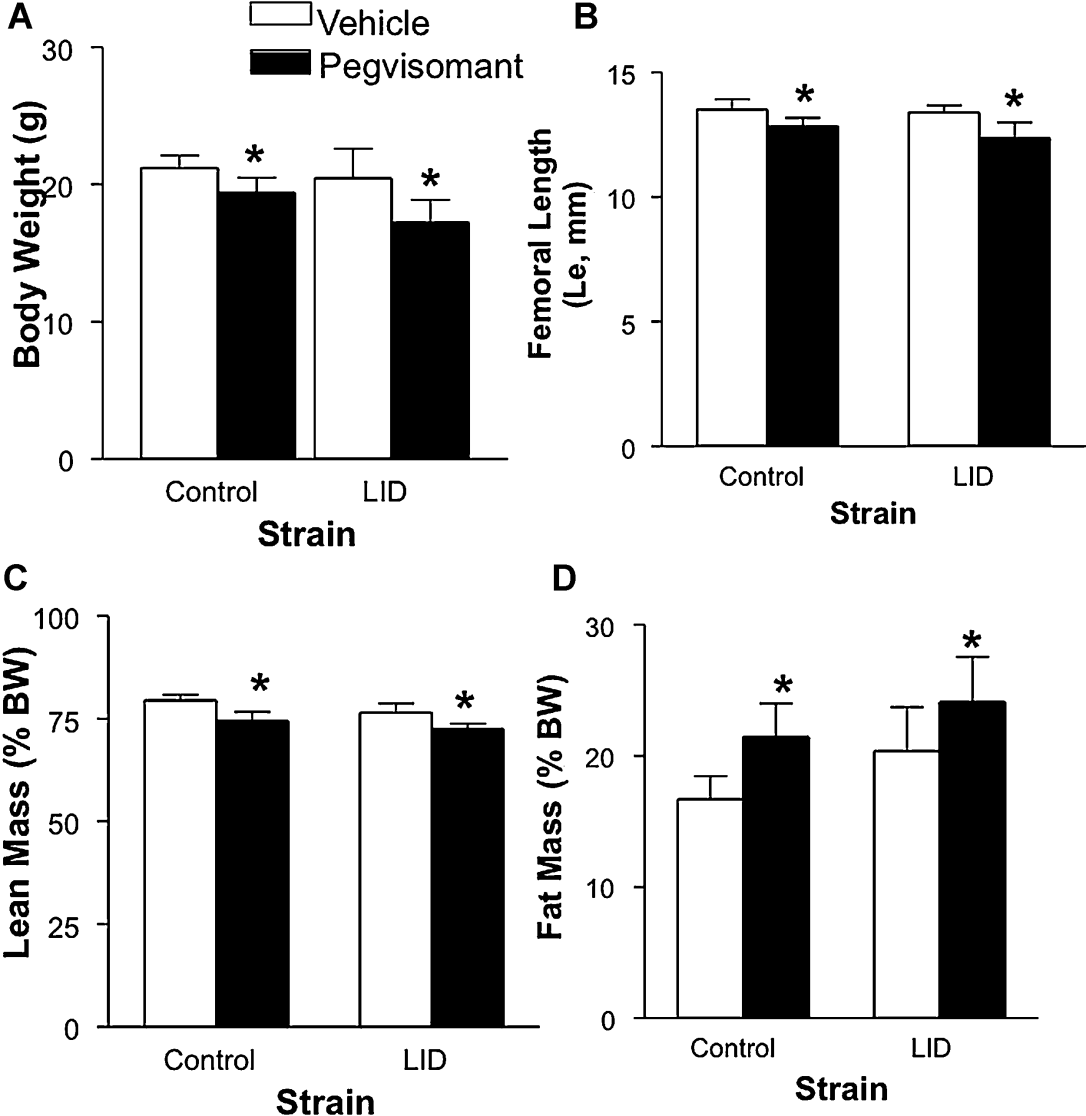
Mean values for (*A*) body weight, (*B*) femur length, (*C*) relative lean mass, and (*D*) relative fat mass (± SD) for 8-week-old control and LID mice treated with vehicle (*n* = 15 and *n* = 16, respectively) or pegvisomant (*n* = 10 and *n* = 11, respectively). ^*^Significant difference between vehicle and pegvisomant treatment, ANOVA, *p* < .05.

In both control and LID mice, significant reductions in body weight and femoral length were observed after treatment with pegvisomant (Fig. 3*A, B*). For mean body weight, the reduction in pegvisomant-treated LID mice (approximately 15%) was significantly greater than the reduction in control mice (approximately 8%). However, the reduction in mean femoral length (Le) owing to pegvisomant was not significantly different between LID (approximately 7%) and control mice (approximately 5%). Quantification of body composition by MRI revealed that the reduction in body weight was largely a result of a significant decrease in absolute lean mass (ANOVA, *p* < .05) for both control (16.86 g before, 14.43 g after) and LID (15.62 g before, 12.47 g after) mice. Changes in absolute fat mass after pegvisomant treatment were not significantly different for control (3.54 g before, 5.18 g after) and LID (4.23 g before, 4.20 g after). Consequently, for both control and LID mice, the percentage of lean mass relative to body weight decreased significantly (Fig. 3*C*), as expected owing to inhibition of GH, and the percentage of fat mass relative to body weight increased significantly (Fig. 3D) as a result of pegvisomant treatment.

### Pharmacologic inhibition of GH action during puberty leads to impaired bone accrual

In nearly all cases, the mid-diaphyseal cortical bone traits affected by pegvisomant treatment were the same for control and LID mice (Table 1). Ct.Ar, Ct.Th, RCA, and *J*_*o*_ all were decreased significantly in control and LID mice, indicating significant reductions in the amount of bone tissue (reduced Ct.Ar, Ct.Th, and RCA), as well as a distribution of bone tissue closer to the center of the marrow cavity (reduced *J*_*o*_). Tt.Ar values also were reduced in both strains, but the reduction did not reach statistical significance in the control mice. Ma.Ar, robustness, and tissue mineral density (TMD) all were unchanged in both control and LID mice treated with pegvisomant. Finally, for all cortical bone traits (Ct.Ar, Tt.Ar, Ma.Ar, *J*_*o*_, Ct.Th, and TMD) and derived traits (robustness and RCA), the mean values of pegvisomant-treated control mice were statistically indistinguishable from those of vehicle-treated LID mice (ANOVA, *p* < .05; Table 1).

**Table 1:** Mean Cortical Bone Traits and Derived Traits (± SD) for 8-Week-Old Male Control and LID Mice Treated With Vehicle or Pegvisomant

Control	Vehicle *n* = 15	Pegvisomant *n* = 10
Tt.Ar (mm^2^)	1.25 ± 0.11	1.15 ± 0.08
Ct.Ar (mm^2^)*	0.64 ± 0.05	0.56 ± 0.04
Ma.Ar(mm^2^)	0.61 ± 0.07	0.59 ± 0.04
Jo (mm^4^)*	0.20 ± 0.03	0.16 ± 0.02
Ct.Th (mm)*	0.17 ± 0.01	0.16 ± 0.01
TMD (mg/cc)	1221 ± 28	1232 ± 33
Robustness (Tt.Ar/Le)	0.092 ± 0.007	0.090 ± 0.005
RCA (Ct.Ar/Tt.Ar)*	0.513 ± 0.023	0.486 ± 0.013

LID	Vehicle *n* = 16	Pegvisomant *n* = 11
Tt.Ar (mm^2^)*	1.12 ± 0.12	1.00 ± 0.12
Ct.Ar (mm^2^)*	0.56 ± 0.07	0.47 ± 0.05
Ma.Ar(mm^2^)	0.57 ± 0.07	0.53 ± 0.08
Jo (mm^4^)*	0.16 ± 0.04	0.12 ± 0.03
Ct.Th (mm)*	0.16 ± 0.01	0.14 ± 0.01
TMD (mg/cc)	1221 ± 37	1210 ± 35
Robustness (Tt.Ar/Le)	0.084 ± 0.008	0.081 ± 0.010
RCA (Ct.Ar/Tt.Ar)*	0.497 ± 0.020	0.467 ± 0.022

* Significant difference between vehicle and pegvisomant treatment, ANOVA, *p* < .05.

Pegvisomant treatment resulted in only minor changes in the trabecular bone of control mice, as evidenced by a small increase in Tb.Th and trabecular TMD (Table 2). The relative amount of bone tissue present (BV/TV) was unchanged, as was the number and spacing of trabeculae. For LID mice, significant reductions were seen in Tb.N, and as a result, Tb.Sp was increased significantly. Tb.Th was unchanged in pegvisomant-treated LID mice, and as in control mice, BV/TV and trabecular TMD were unchanged.

**Table 2:** Mean Trabecular Bone Traits (± SD) for 8-Week-Old Male Control and LID Mice Treated with Vehicle or Pegvisomant

Control	Vehicle *n* = 14	Pegvisomant *n* = 10
BV/TV (%)	14.7 ± 2.7	15.1 ± 1.6
Tb.Th (mm)*	0.028 ± 0.002	0.032 ± 0.001
Tb.N (mm^−1^)	5.3 ± 0.5	4.8 ± 0.4
Tb.Sp(mm)	0.163 ± 0.021	0.180 ± 0.020
TMD (mg/cc)*	590 ± 37	664 ± 40

LID	Vehicle *n* = 16	Pegvisomant *n* = 11
BV/TV (%)	16.7 ± 5.0	12.6 ± 5.3
Tb.Th (mm)	0.029 ± 0.003	0.027 ± 0.003
Tb.N (mm^−1^)*	5.8 ± 1.2	4.5 ± 1.4
Tb.Sp (mm)*	0.153 ± 0.045	0.215 ± 0.072
TMD (mg/cc)	638 ± 49	607 ± 40

* Significant difference between vehicle and pegvisomant treatment, ANOVA, *p* < .05.

**Fig. 4:**
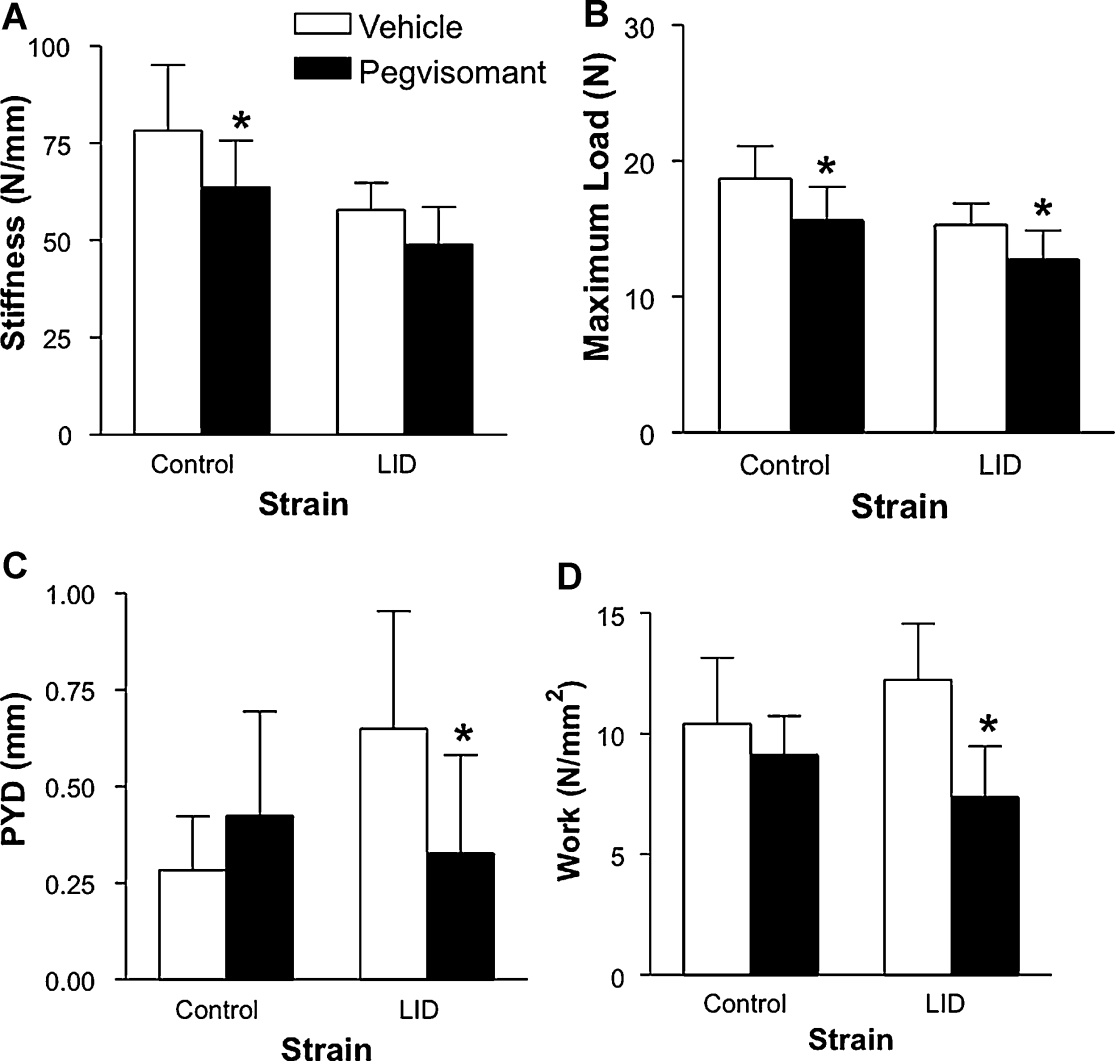
Mean values for (*A*) stiffness, (*B*) maximum load, (*C*) PYD, and (*D*) work (± SD) for 8-week-old control and LID mice treated with vehicle (*n* = 15 each) or pegvisomant (*n* = 10 and *n* = 11, respectively). ^*^Significant difference between vehicle and pegvisomant treatment, ANOVA, *p* < .05.

Mechanical properties of femurs from treated mice were assessed by four-point bending and correlated with bone morphologic traits obtained by µCT. Control mice demonstrated significant reductions in both stiffness and maximum load after treatment with pegvisomant (Fig. 4*A, B*). In LID mice, the reduction in stiffness did not reach significance, but maximum load was reduced significantly. PYD and work also were reduced significantly in LID mice but not in control mice (Fig. 4*C, D*).

### Pharmacologic inhibition of GH action during puberty hinders bone formation

Serum levels of the bone-formation marker osteocalcin were reduced significantly in pegvisomant-treated control mice compared with controls (Fig. 2*B*) but did not reach significance in pegvisomant-treated LID mice. Mean values for L.Pm, MAR, and BFR on the endosteal surface of the femoral midshaft were statistically indistinguishable among all groups (no differences were found when comparing strains or treatment groups) (Table 3). However, when comparing periosteal groups, although L.Pm and MAR did not differ, mean BFR was significantly lower in pegvisomant-treated control and LID mice than in their respective vehicle-treated counterparts. This was due to lower BFR values for animals that had double labels, as well as an increase in the number of animals that had no double labeling as a result of pegvisomant treatment. Mean values for periosteal BFR in pegvisomant-treated LID and control mice also were significantly smaller than the mean BFR for all endosteal groups. Similar surface differences were observed in L.Pm, which was significantly smaller on the periosteal surface of pegvisomant-treated animals than on the endosteal surface of these animals.

**Table 3:** Mean (±SD) Histomorphometry Data From Mid-diaphyseal Cortical Bone of 8-Week-Old Male Control (*n* = 9/Group) and LID (*n* = 7/Group) Mice Treated With Vehicle or Pegvisomant

	L. Pm (%)	MAR (µm/day)	BFR/B. Pm (µm/day*100)	#Samples Lacking Double Labels
Periosteal surface				
Control (Vehicle)	30.8 ± 8.9^b^	2.8 ± 1.3	91.1 ± 46.3^d^	1
Control (Pegvisomant)	22.1 ± 11.4^b^	1.6 ± 1.1	45.9 ± 32.2^c^	2
LID (Vehicle)	29.6 ± 14.1	2.5 ± 1.2	85.7 ± 44.9^e^	1
LID (Pegvisomant)	25.6 ± 12.4^b^	1.6 ± 1.6	57.3 ± 55.3^c^	3
Endosteal surface				
Control (Vehicle)	60.1 ± 12.2	2.3 ± 0.7	136.2 ± 47.2	0
Control (Pegvisomant)	71.6 ± 12. 7	2.0 ± 0.4	146.5 ± 51.5	0
LID (Vehicle)	72.4 ± 12.5	2.6 ± 0.4	189.1 ± 47.9	0
LID (Pegvisomant)	98.4 ± 68.3^a^	2.2 ± 0.9	176.0 ± 53.1	0

Statistical differences (ANVOVA, *p* < .05) are indicated by superscript letters.

a Significantly different from all periosteal groups.

b Significantly different from endosteal LID (Vehicle).

c Significantly different for all other edosteal and perisosteal groups.

d Significantly different from endosteal LID(Vehicle).

e Significantly different from periosteal Control (Pegvisomant).

## Discussion

Our data indicate a role for GH in establishing pubertal skeletal and body size that is independent of hepatic IGF-1 production. We show that treatment with the growth hormone antagonist pegvisomant resulted in significant decreases in body weight, femur length, and skeletal acquisition in control mice but even more prominent reductions in mice with reduced serum IGF-1 (LID mice). Specifically, inhibition of GH action resulted in significant reductions in lean mass relative to body weight for both groups, which was expected given that GH is known to promote muscle and bone growth.(25–28) In LID mice, where serum IGF-1 levels were about 25% of control levels, the decrease in body weight owing to pegvisomant treatment was more severe. These findings are in agreement with a prior study that showed that *Ghr/Igf1* nullizygotes have more severe growth retardation than mice with single ablations of either the *Ghr* or the *Igf1* gene.(29)

**Fig. 5:**
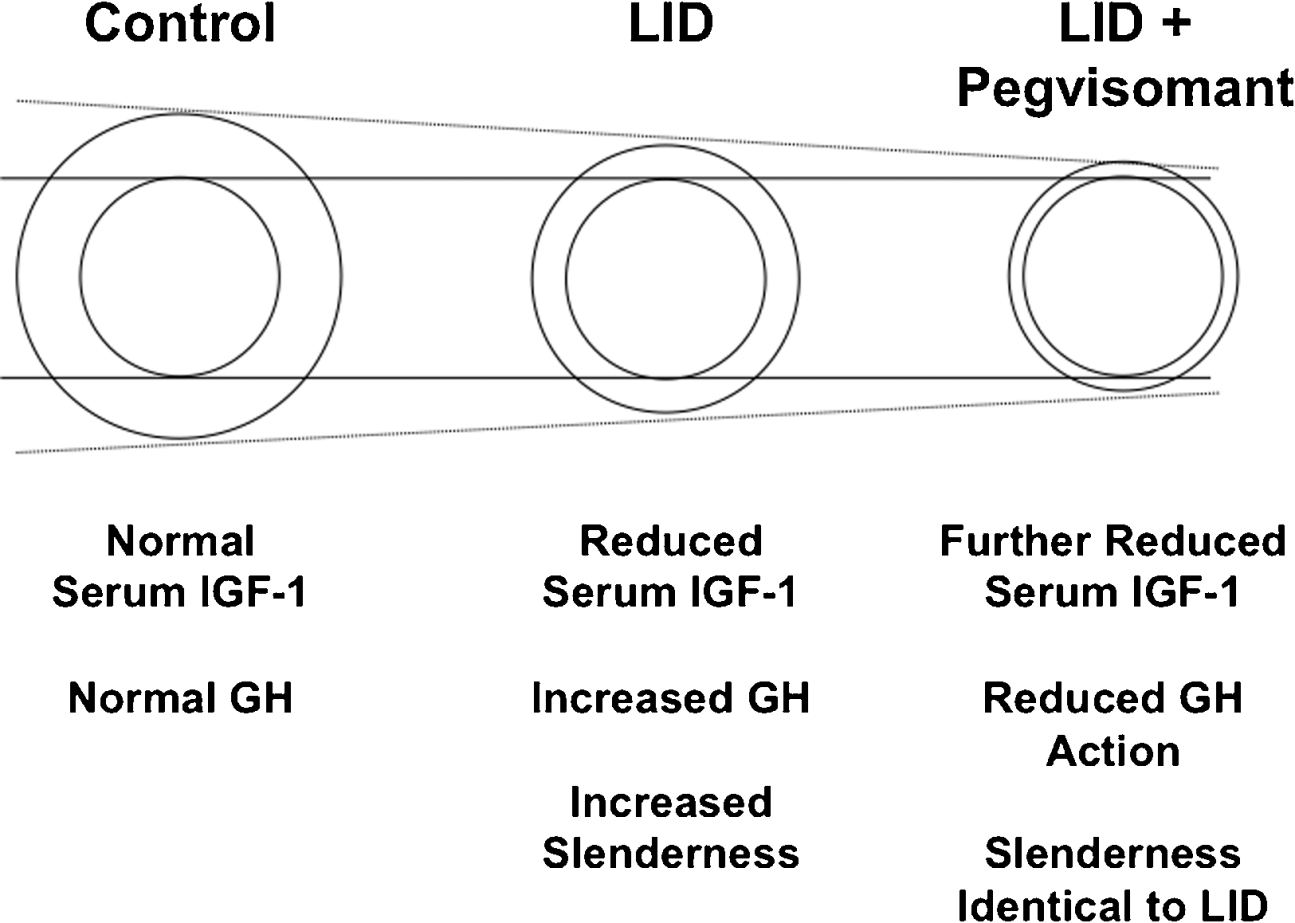
Schematic representation of mid-diaphyseal cortical bone from 8-week-old male mice after alterations in serum IGF-1 and GH levels. The dotted lines indicate a reduction in Tt.Ar as a result of loss of IGF-1 and GH action. Solid lines indicate no alteration in Ma.Ar as a result of diminished IGF-1 or GH action. Reduction in serum IGF-1 (LID mouse) results in a smaller, more slender bone compared with control mice by 8 weeks. Reduction in GH action at 4 weeks results in further abrogation of skeletal development in LID mice by 8 weeks such that the femurs are smaller. However, size reductions in femur cross-sectional area are matched to reductions in femur length such that GH blockade does not result in bones that are more slender.

While inhibition of GH action in control mice during puberty resulted in decreases in serum IGF-1 levels, as described previously,(17) in LID mice, in which the liver *Igf1* gene was ablated, treatment with pegvisomant decreased serum IGF-1 levels an additional 47%. Following treatment with pegvisomant, control mice had significant reductions in cortical bone traits (Ct.Ar, *J*_*o*_, Ct.Th, and RCA; Table 1). Likewise, when LID mice were treated with pegvisomant, further reductions in cortical bone traits were observed, resulting in an even smaller cross-sectional size (Tt.Ar and *J*_*o*_), less total bone tissue (Ct.Ar), and less bone tissue relative to bone area (RCA). In addition, femur length (Le) was reduced significantly in pegvisomant-treated LID mice, indicating reduced GH action in the femoral growth plate. Interestingly, these reductions in femur length (Le) were proportionally matched to reductions in transverse bone size such that pegvisomant-treated LID mice (as well as control mice) had identical robustness values when compared with vehicle-treated animals. Thus loss of GH action during puberty appears to hinder both transverse and longitudinal bone growth in a coordinated fashion, whereas loss of hepatic IGF-1, as reported previously in the LID mouse,(4) reduces transverse bone growth but not longitudinal growth, thereby resulting in a less robust, more slender phenotype for LID mice (Fig. 5).

Reductions in cortical bone size and amount of bone tissue in both control and LID mice treated with pegvisomant were substantial, and this suggested that the bones would have reduced mechanical properties. Testing under four-point bending confirmed this hypothesis. Control mice treated with pegvisomant had significantly reduced femur stiffness and maximum load values. LID mice, which are at a mechanical disadvantage to begin with, had even greater reductions in maximum load, PYD, and work when treated with pegvisomant. Given that reductions in PYD are often associated with variations in bone tissue composition,(30,31) we measured cortical TMD values. However, no differences were found as a result of antagonist treatment in LID mice. Still, other factors such as mineral composition, mineral maturity, and collagen content may have been altered by the dual reductions in serum IGF-1 and GH action, and this will require further investigation.

For both control and LID mice, inhibition of GH signaling resulted in no changes to Ma.Ar, suggesting that reductions in transverse bone size (Tt.Ar and *J*_*o*_) were a result of inhibition of periosteal bone apposition, not endosteal bone resorption. This was further supported by our histomorphometric data, which showed reduced L.Pm and BFR on the periosteal surfaces of pegvisomant-treated control and LID mice compared with the endosteal surfaces of these mice, which were unaffected by pegvisomant treatment. It should be noted that inhibition of periosteal surface bone dynamics has been reported previously in untreated LID mice (reduced serum IGF-1 levels)(4) and in *Als* knockout (ALSKO) mice.(32) Taken together, these data indicate a surface-specific effect of the GH/IGF-1 axis during bone accrual.

In summary, our data suggest that in cases where hepatic IGF-1 is diminished, elevations in GH may protect against a severe inhibition of bone modeling during growth in an IGF-1-dependent or -independent mechanism. Blockade of compensatory increases in GH in LID mice led to further decreases in bone size and tissue amount, which resulted in the formation of bones that may be too weak for normal or extreme loading conditions. Thus GH appears important for matching tissue amount (Ct.Ar) to bone size (Tt.Ar), bone length (Le) and body size during puberty.
